# [Retracted] Twist induces epithelial-mesenchymal transition in cervical carcinogenesis by regulating the TGF-β/Smad3 signaling pathway 

**DOI:** 10.3892/or.2025.8960

**Published:** 2025-07-28

**Authors:** Qiong Fan, Mei-Ting Qiu, Zhu Zhu, Jin-Hua Zhou, Limo Chen, Ye Zhou, Wei Gu, Li-Hua Wang, Zhu-Nan Li, Ying Xu, Wei-Wei Cheng, Dan Wu, Wei Bao

Oncol Rep 34: 1787–1794, 2015; DOI: 10.3892/or.2015.4143

Following the publication of this paper, it was drawn to the Editor's attention by a concerned reader that, for the Transwell migration and invasion assay experiments shown in Fig. 3C and D on p. 1791, two pairs of the data panels appeared to be overlapping, suggesting that the same data may have been used in this figure to represent data that were intended to show the results from differently performed experiments. Although the authors responded to the original emailed request from the Editorial Office concerning these data and repeated the experiments shown in this figure, upon performing an independent analysis of the original (published) figures in the office, it came to light that Figs. 1 and 4 also contained probable anomalies. In Fig. 1C and D, there were cells/small areas of the images located in the bottom left hand corners of the images that were strikingly similar, to the extent that this would have been difficult to attribute to coincidence. Furthermore, in the case of Fig. 4, the gel slices in Fig. 4B showed evidence of possible cutting-and-splicing events.

Owing to the fact that these probable anomalies were also identified that could have affected the interpretation of two additional figures in the published article, the Editor of *Oncology Reports* has decided that this paper should be retracted from the Journal due to a lack of confidence in the presented data. The authors were asked for an explanation to account for these concerns, but the Editorial Office did not receive a satisfactory reply. The Editor apologizes to the readership for any inconvenience caused.

